# Exploring the Effect of Differentially Expressed Long Non-coding RNAs Driven by Copy Number Variation on Competing Endogenous RNA Network by Mining Lung Adenocarcinoma Data

**DOI:** 10.3389/fcell.2020.627436

**Published:** 2021-01-28

**Authors:** Huihui Hu, Hangdi Xu, Fen Lu, Jisong Zhang, Li Xu, Shan Xu, Hanliang Jiang, Qingxin Zeng, Enguo Chen, Zhengfu He

**Affiliations:** ^1^Department of Respiratory, College of Medicine, Sir Run Run Shaw Hospital, Zhejiang University, Hangzhou, China; ^2^Operation Room, College of Medicine, Sir Run Run Shaw Hospital, Zhejiang University, Hangzhou, China; ^3^Department of Thoracic Surgery, College of Medicine, Sir Run Run Shaw Hospital, Zhejiang University, Hangzhou, China

**Keywords:** lncRNA, copy number variation, TCGA, LUAD, ceRNA network

## Abstract

Lung cancer is the first cause of cancer death, and gene copy number variation (CNV) is a vital cause of lung cancer progression. Prognosis prediction of patients followed by medication guidance by detecting CNV of lung cancer is emerging as a promising precise treatment in the future. In this paper, the differences in CNV and gene expression between cancer tissue and normal tissue of lung adenocarcinoma (LUAD) from The Cancer Genome Atlas Lung Adenocarcinoma data set were firstly analyzed, and greater differences were observed. Furthermore, CNV-driven differentially expressed long non-coding RNAs (lncRNAs) were screened out, and then, a competing endogenous RNA (ceRNA) regulatory network related to the gene CNV was established, which involved 9 lncRNAs, seven microRNAs, and 178 downstream messenger RNAs (mRNAs). Pathway enrichment analyses sequentially performed revealed that the downstream mRNAs were mainly enriched in biological pathways related to cell division, DNA repair, and so on, indicating that these mRNAs mainly affected the replication and growth of tumor cells. Besides, the relationship between lncRNAs and drug effects was explored based on previous studies, and it was found that LINC00511 and LINC00942 in the CNV-associated ceRNA network could be used to determine tumor response to drug treatment. As examined, the drugs affected by these two lncRNAs mainly targeted metabolism, target of rapamycin signaling pathway, phosphatidylinositol-3-kinase signaling pathway, epidermal growth factor receptor signaling pathway, and cell cycle. In summary, the present research was devoted to analyzing CNV, lncRNA, mRNA, and microRNA of lung cancer, and nine lncRNAs that could affect the CNV-associated ceRNA network we constructed were identified, two of which are promising in determining tumor response to drug treatment.

## Introduction

Lung cancer is the leading cause of cancer death in modern times, and it has caused 140,000 deaths in the first three quarters of 2020, and ~220,000 people have suffered from lung adenocarcinoma (LUAD), according to the Surveillance, Epidemiology, and End Results database of the National Cancer Institute (Travert et al., 2020). Lung cancer has brought a great burden on patients, their families, and the whole society. Treatments for lung cancer mainly include surgical resection, radiotherapy, chemotherapy, and drug therapy (Lai et al., 2019; Leonetti et al., 2019; Yamada et al., 2019). LUAD is a common type of lung cancer, and surgical resection turns out to be the main treatment. Nevertheless, due to the characteristics of easy metastasis and difficulty in the radical cure, relapses often occur after surgery, and postoperative chemotherapy is hence needed for effective cancer control (Fedor et al., 2013; Salazar et al., 2017). At present, many drugs have been used for the treatment of LUAD, such as Osimertinib, a drug that targets epidermal growth factor receptor, and Brigatinib, which targets anaplastic lymphoma kinase (Mok et al., 2017; Camidge et al., 2018). In addition to targeted drugs, immune drugs are also emerging and becoming popular therapeutic drugs, such as Nivolumab and Ipilimumab (Yang et al., 2018; Pinto et al., 2019). However, due to various reasons, the control effect of these drugs on cancer is often not sustainable, and LUAD cells are prone to develop resistance to these drugs (Xia et al., 2019; Zhu X. et al., 2019). Therefore, looking for factors that affect drug treatment to improve drug treatment effect and prolong the effective inhibition of cancer with drugs is a possible solution to improve the prognosis of patients with LUAD.

Long non-coding RNAs (lncRNAs) are a kind of non-coding RNA that exist in the cytoplasm with a length exceeding 200 bp (Perkel, 2013). LncRNAs, mainly produced by the interruption of protein-coding gene structure, exist in the cytoplasm and interact with other molecules in cells, thereby regulating the physiological and biochemical processes in organism (Fan and Hu, 2016). Existing studies found that lncRNAs can also be encapsulated in exosomes and secreted to the outside of cells, and lncRNAs can also remotely regulate the physiological and biochemical functions of other cells, thereby affecting the body's physiological functions (Dragomir et al., 2018). There are several ways in which lncRNAs affect physiology. For instance, lncRNAs can directly regulate gene expression through influencing RNA synthetase recruitment. In addition, lncRNAs can improve translation efficiency by maintaining mRNA activity and reduce translation speed by colliding ribosomes. Most importantly, lncRNAs can specifically bind with and sponge microRNAs (miRNAs) to block the miRNA-induced degradation of mRNAs (Long et al., 2017). Because cancer drugs often achieve their therapeutic effects by regulating the expression of mRNAs in cells, lncRNAs, which can also affect mRNA expression, are considered to be important factors affecting the therapeutic effect of drugs (Guo et al., 2018). A current study finds that lncRNA small nucleolar RNA host gene three can induce Sorafenib resistance through the miR-128/CD151 pathway (Zhang et al., 2019). Besides, lncRNA HOX antisense intergenic RNA can also affect the effect of medication on lung cancer patients through Wnt signaling pathway (Guo et al., 2018). Therefore, it is believed that the identification of lncRNAs that may affect the effect of drug treatment in LUAD is of great significance to improve the treatment and prognosis of LUAD patients.

DNA copy number variation (CNV), different from other variations, such as insertion, deletion, and dislocation, is a type of variation that results in an increase or a reduction of DNA copy number (Liang et al., 2016). Because DNA copy number is related to the expression of DNA-coding RNA, CNV is considered to be a factor responsible for the alteration in coding RNA expression (Liu et al., 2019). Present studies believe that copy number amplification can greatly elevate the expression of corresponding DNA-coding RNA, whereas copy number deletion will reduce the expression of the coding RNA (Liu et al., 2019). Currently, numerous studies that focus on the CNV of CNV-driven mRNA uncover that CNV of the mRNA can indeed affect prognosis and drug resistance of cancer, and it is reported that the copy number deletion of 17q22 can make prostate cancer develop resistant to Enzalutamide (Lu et al., 2014; Guan et al., 2020). With the understanding regarding the regulatory mechanism of lncRNA going deeper, it is found that DNA CNV of lncRNA is also one of the reasons that drive cells to show differences at the molecular level (Zheng et al., 2020). Additionally, given credit to the development of sequencing technology and bioinformatics methods, detection of CNVs in cancer tissue has become inexpensive and simple, and it has become a feasible detection method to determine the prognosis of patients and the effects of drug treatment.

In this study, CNV, lncRNAs, miRNAs, and mRNAs in The Cancer Genome Atlas Lung (TCGA)-LUAD data set were analyzed to explore a CNV-associated competing endogenous RNA (ceRNA) network related to cancer development, and lncRNAs related to drug treatment of LUAD patients were further explored. LUAD-related data were firstly downloaded from TCGA database, and differential analysis was performed to screen out genes and CNVs with significant differences between normal and tumor tissue. Then, CNV-driven lncRNAs, along with related miRNAs and mRNAs, were screened out. Furthermore, a ceRNA regulatory network based on the identified lncRNAs, miRNAs, and mRNAs was established, and the key lncRNAs and mRNAs were identified. Finally, the molecular biological functions that might be affected by the network mRNAs were analyzed, and lncRNAs related to drug treatment in LUAD were investigated.

## Materials and Methods

### Data Downloading

**Figure 1:**
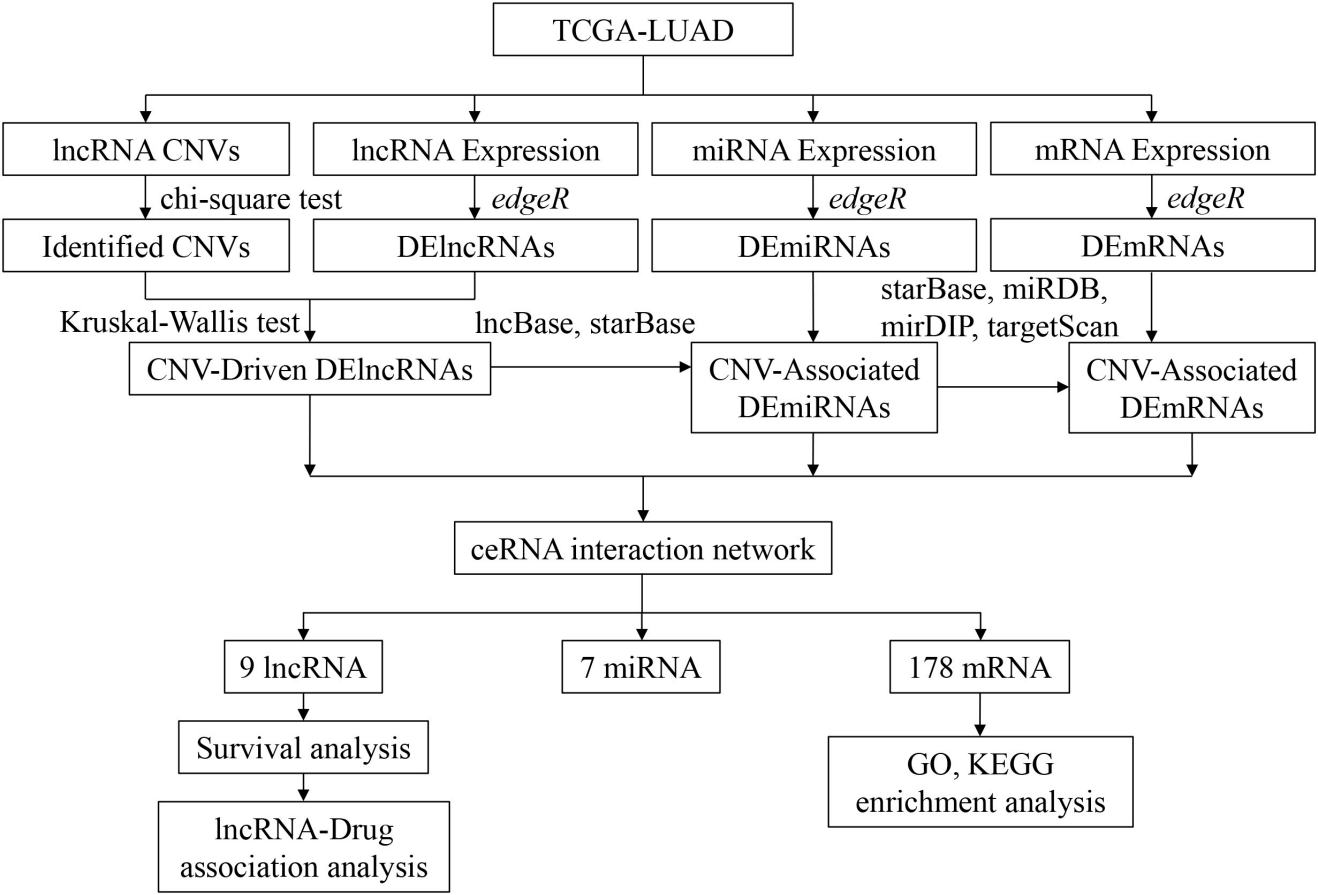
Overall workflow of this study.

Transcriptome expression data (normal: 59, tumor: 535), mature miRNA expression data (normal: 46, tumor: 521), and DNA copy number data (normal: 591, tumor: 556) of LUAD were downloaded from TCGA database. According to the annotation of the human genome GRCh38, corresponding mRNA and lncRNA expression data were extracted from the transcriptome expression data. Corresponding clinical data were downloaded on September 23, 2020, in the meanwhile (Supplementary Table 1). To explain the analytic process more clearly, a flow chart of this study was drawn (Figure 1).

### Differential Analysis

Samples were divided into the tumor or normal group. R package edgeR was applied to perform differential analysis to filter differentially expressed lncRNAs, miRNAs, and mRNAs (DElncRNAs, DEmiRNAs, and DEmRNAs), with |logFC| > 1.5 and FDR < 0.05 as thresholds.

### Identification of Copy Number Variation-Driven Long Non-coding RNAs

The chi-square test was used to analyze whether there is a significant difference in the CNV of lncRNA in normal tissue samples and tumor samples. The Bonferroni method was used to correct the *p-*value and lncRNAs with CNVs with adjusted. *p* < 0.05 were screened. lncRNAs with copy number amplification and with differentially upregulated expression were intersected, whereas lncRNAs with copy number deletion and with differentially downregulated expression were intersected. Then, the Kruskal–Wallis test was used to analyze the correlation between the CNV and the differential expression to select CNV-driven DElncRNAs (*p* < 0.05).

### Construction of Copy Number Variation-Driven Competing Endogenous RNA Network

miRNAs that interacted with CNV-driven DElncRNAs were predicted through LncBase database (http://carolina.imis.athena-innovation.gr/diana_tools/web/index.php?r=lncbasev2%2Findex) and starBase database (http://starbase.sysu.edu.cn/index.php), and the predicted miRNAs were then intersected with DEmiRNAs to select the DEmiRNAs that interacted with CNV-driven DElncRNAs. Pearson correlation analysis was conducted to calculate the correlation between the expression of CNV-driven DElncRNAs and the DEmiRNAs mentioned earlier. The DEmiRNAs with *r* < −0.2 and *p* < 0.05 were selected as downstream CNV-associated DEmiRNAs regulated by lncRNAs.

Similarly, mRNAs that had an interactive relationship with CNV-associated DEmiRNAs (the mRNAs in at least two databases were considered to have an interactive relationship with CNV-associated DEmiRNAs) were predicted through starBase, miRDB (http://mirdb.org/), mirDIP (http://ophid.utoronto.ca/mirDIP/index.jsp), and TargetScan (http://www.targetscan.org/vert_72/) databases. The predicted mRNAs mentioned earlier were intersected with DEmRNAs, and the DEmRNAs with *r* < −0.2 and *p* < 0.05 were regarded as downstream CNV-associated DEmRNAs regulated by miRNAs.

Based on the results mentioned earlier, lncRNAs, miRNAs, and mRNAs with complete regulatory relationships were selected to establish a corresponding ceRNA network for subsequent analysis. The ceRNA network was the CNV-associated ceRNA network visualized by the Cytoscape, and the R package GISTIC2 was used to draw a chromosomal map showing CNV regions in coding genes of lncRNAs. Afterward, patients were divided into Amplification group, Deletion group, and Diploid group according to the CNV of lncRNAs in the ceRNA network. Box plots were used to show the relationship between CNV and lncRNA expression.

### Kyoto Encyclopedia of Genes and Genomes and Gene Ontology Enrichment Analyses

Based on the mRNAs in the ceRNA network, R package ClusterProfiler was used to perform Gene Ontology annotation and Kyoto Encyclopedia of Genes and Genomes pathway enrichment analysis to confirm the biological functions and signaling pathways that might be affected by the mRNAs in the ceRNA network.

### Survival Analysis

For survival analysis, patients were grouped according to the CNV of corresponding lncRNAs. R package survival was used to draw survival curves of patients to evaluate the possible impact of CNV of lncRNAs on the prognosis.

## Results

### Differential Analysis

**Figure 2:**
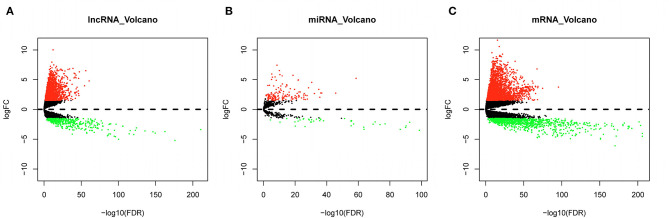
Differential analysis results. **(A)** Volcano plot of DElncRNAs; **(B)** volcano plot of DEmiRNAs; **(C)** volcano plot of DEmRNAs; green dots represent differentially downregulated genes, and red dots represent differentially upregulated genes.

Expression data of lncRNA, miRNA, and mRNA of LUAD were obtained from TCGA database, samples were grouped into normal or tumor sample, and differential analysis was performed. Finally, a total of 2,555 DElncRNAs, including 2,142 upregulated lncRNAs and 413 downregulated lncRNAs, were obtained from lncRNA data (Figure 2A). A total of 186 DEmiRNAs, including 147 upregulated miRNAs and 39 downregulated miRNAs, were obtained from miRNA data (Figure 2B). A total of 3,591 DEmRNAs, including 2,553 upregulated mRNAs and 1,038 downregulated mRNAs, were obtained from mRNA data (Figure 2C).

### Selection of Copy Number Variation-Driven Long Non-coding RNAs

**Figure 3:**
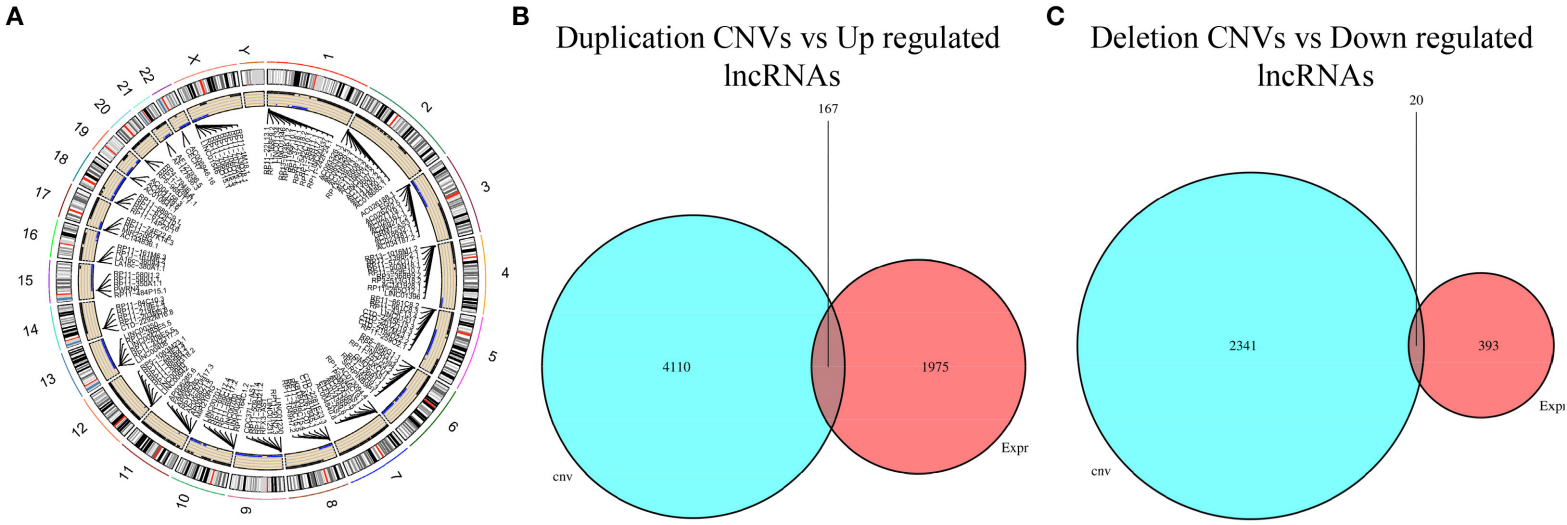
Filtering of CNV-driven DElncRNAs. **(A)** Circle map of lncRNA CNVs in LUAD (black dots represent duplication CNVs, and blue dots represent deletion CNVs; **(B)** Venn diagram filters upregulated lncRNAs with duplication CNVs; **(C)** Venn diagram filters downregulated lncRNAs with deletion CNVs.

To screen CNV-driven lncRNAs, the chi-square test was first used to analyze the difference in CNVs in cancer tissue and normal tissue. The results denoted significant CNVs in coding genes of 6,640 lncRNAs in LUAD tissue (Figure 3A). According to the lncRNA differential expression and CNV, the lncRNAs with consistent alteration in CNV and expression were screened out. Firstly, 167 differentially upregulated lncRNAs with copy number amplification and 20 differentially downregulated lncRNAs with copy number deletion were obtained (Figures 3B,C). Then, the correlation between CNV and lncRNA expression was tested, and finally, 67 CNV-driven DElncRNAs were obtained (Supplementary Table 2).

### Establishment of Competing Endogenous RNA Regulatory Network

**Figure 4:**
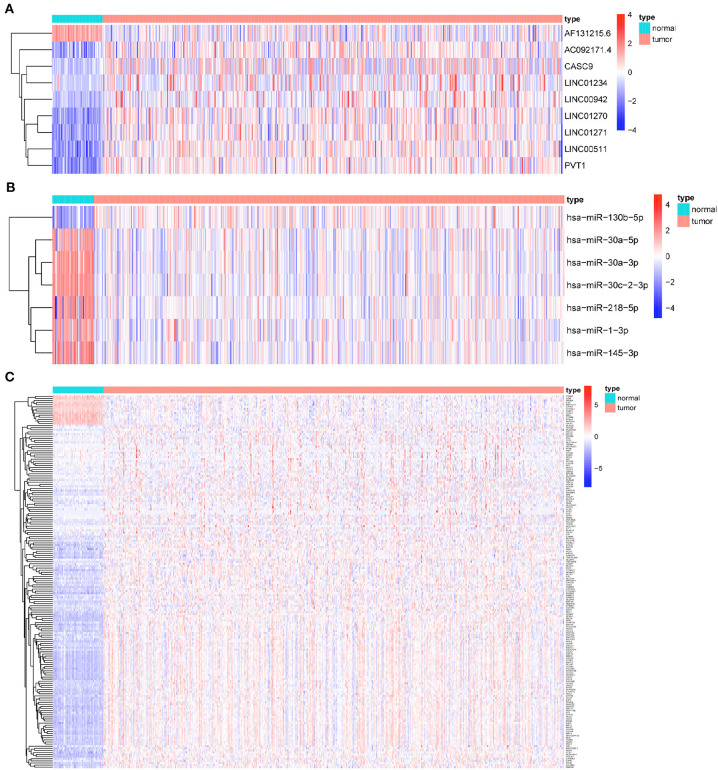
Heatmaps of RNAs in CNV-associated ceRNA network. **(A)** Heatmap of lncRNAs in CNV-associated ceRNA network; **(B)** heatmap of miRNAs in CNV-associated ceRNA network; **(C)** heatmap of mRNAs in CNV-associated ceRNA network.

**Figure 5:**
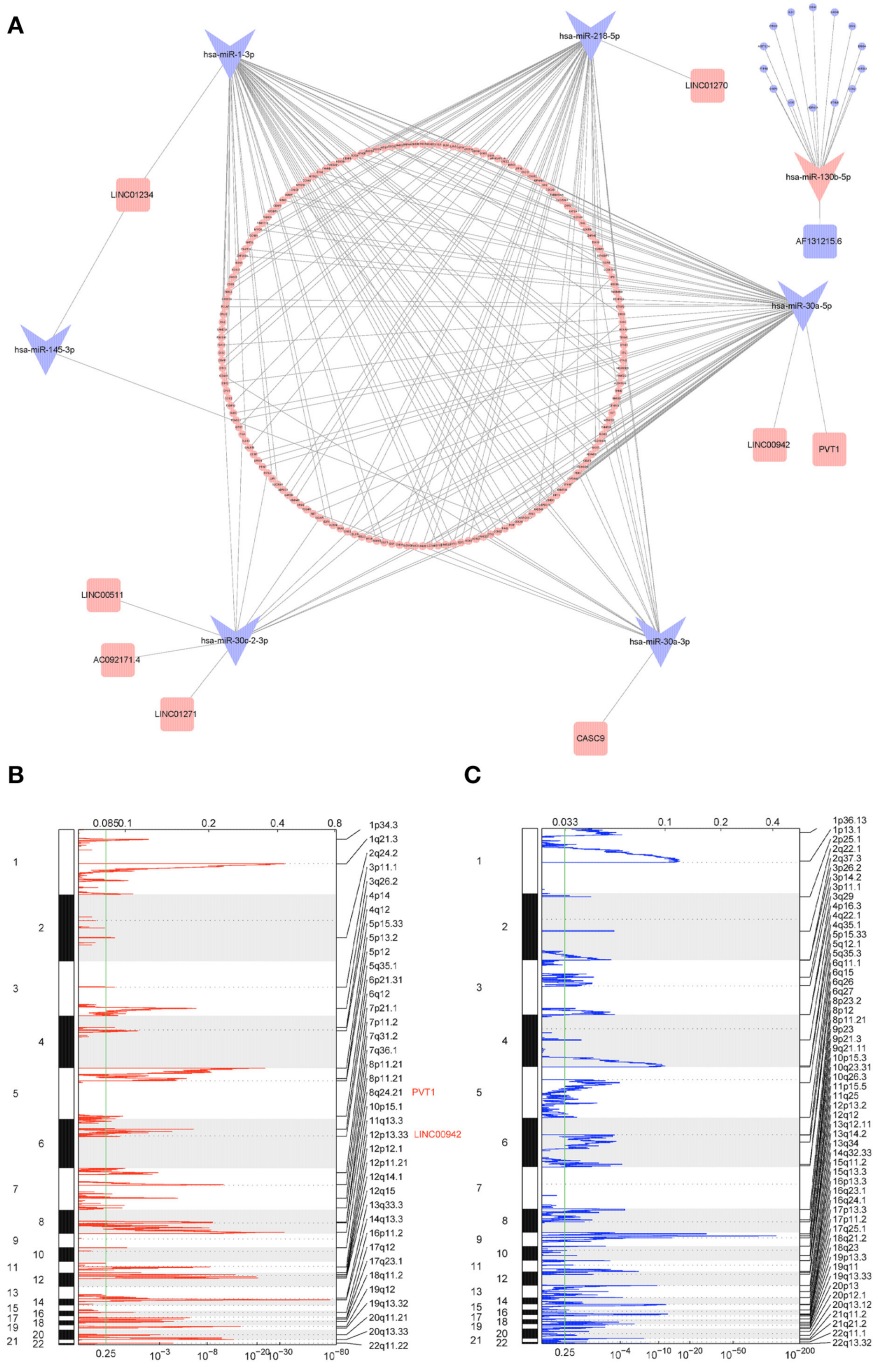
Overview of CNV-associated ceRNA network and CNVs of lncRNAs. **(A)** Interaction between different RNAs in the CNV-associated ceRNA network; the shape of each node represents the type of RNA (squares represent lncRNAs, swallowtail quadrilaterals represent miRNAs, and circles represent mRNAs), and the color of each node represents the regulation type (the red represents upregulated, and the blue represents downregulated); **(B,C)** chromosomal view of amplification and deletion peaks between tumor and normal tissue; G-score (top) and *p*-value (bottom) were calculated by GISTIC2; right axis represents chromosomal position, and examples of CNV-driven DElncRNAs located in the peaks are labeled.

Based on the CNV-driven DElncRNAs we identified earlier, a CNV-associated ceRNA network that contained nine lncRNAs, seven miRNAs, and 178 mRNAs was established through multiple databases (Table 1, Figures 4A–C). The Cytoscape was run to visualize the network to make the gene regulatory relationship more clearly (Figure 5A). Finally, chromosomal maps showing CNV regions were drawn, and some lncRNAs in the CNV-associated ceRNA network were marked (Figures 5B,C).

**Table 1:** Detailed information on CNV-driven DElncRNAs in the CNV-associated ceRNA network.

**Chr**	**Gene**	**Start**	**End**	**Band**
chr7	AC092171.4	5475804	5479811	p22.1
chr8	AF131215.6	11062647	11067089	p23.1
chr8	CASC9	75223404	75324741	q21.13
chr8	PVT1	127794533	128101253	q24.21
chr12	LINC00942	1500525	1507318	p13.33
chr12	LINC01234	113679459	113773683	q24.13
chr17	LINC00511	72323123	72640472	q24.3
chr20	LINC01270	50292720	50314922	q13.13
chr20	LINC01271	50310711	50321342	q13.13

### Pathway Enrichment Analyses of Copy Number Variation-Driven Differentially Expressed Messenger RNAs

**Figure 6:**
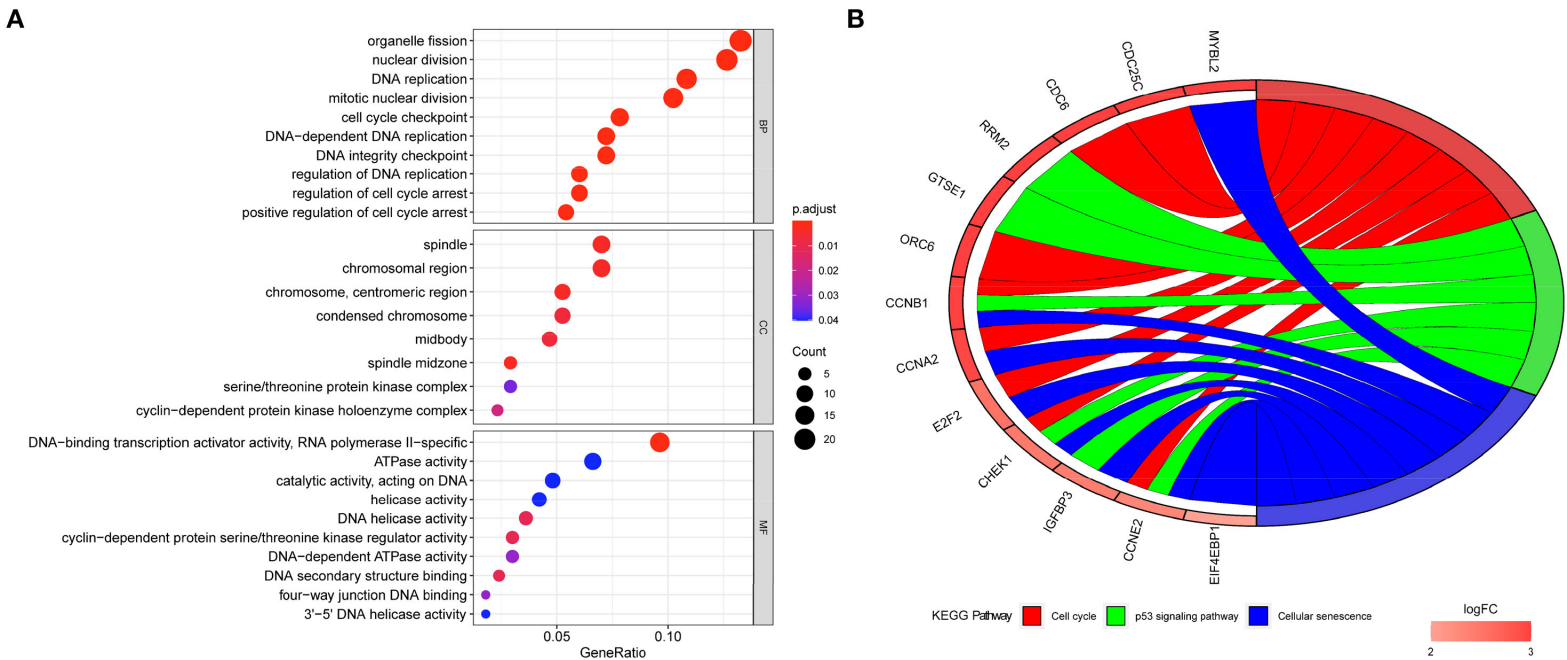
Functional enrichment analyses of mRNAs in CNV-associated ceRNA network. **(A)** Gene Ontology annotation of mRNAs in the CNV-associated ceRNA network; **(B)** Kyoto Encyclopedia of Genes and Genomes pathway enrichment of mRNAs in the CNV-associated ceRNA network.

After the ceRNA network was established, enrichment analyses were conducted on the network mRNAs to explore the biological functions and signaling pathways that might be affected. Gene Ontology annotation analysis revealed that these mRNAs were mainly enriched in biological functions such as organelle fission, nuclear division, DNA replication, mitotic nuclear division, and cell cycle checkpoint (Figure 6A). Kyoto Encyclopedia of Genes and Genomes enrichment analysis exhibited that these genes were mainly enriched in pathways involved in the cell cycle, cellular senescence, and p53 signaling pathway (Figure 6B). These results demonstrated that the mRNAs in the CNV-associated ceRNA network were mainly enriched in pathways related to cell division, DNA repair, and so on.

### Copy Number Variation-Driven Differentially Expressed Long Non-coding RNAs May Affect the Prognosis of Lung Adenocarcinoma Patients

**Figure 7:**
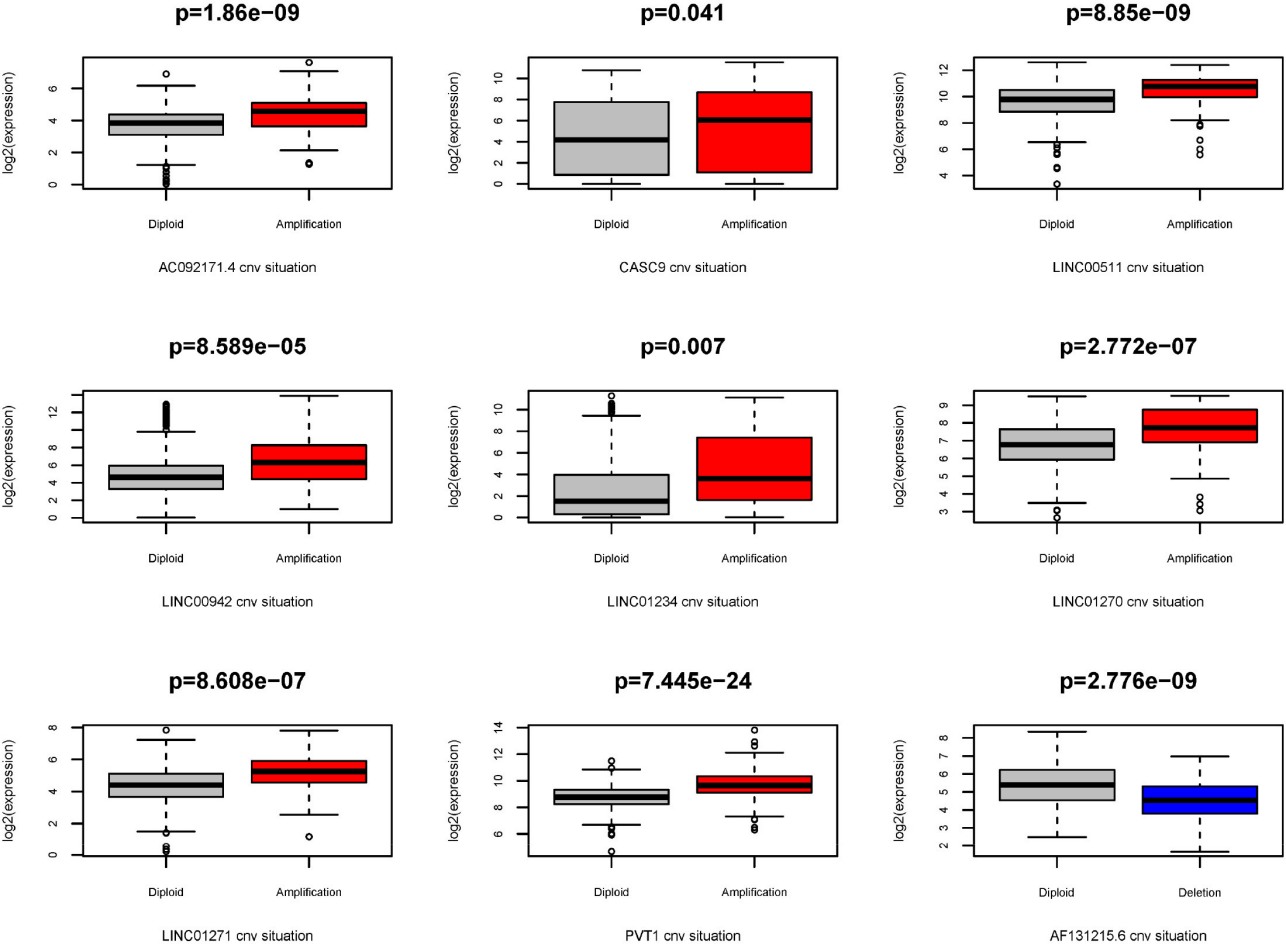
Association between expression and CNV of lncRNAs. Gray box represents the normal group, red box represents patients with amplification CNVs, and blue box represents patients with deletion CNVs.

**Figure 8:**
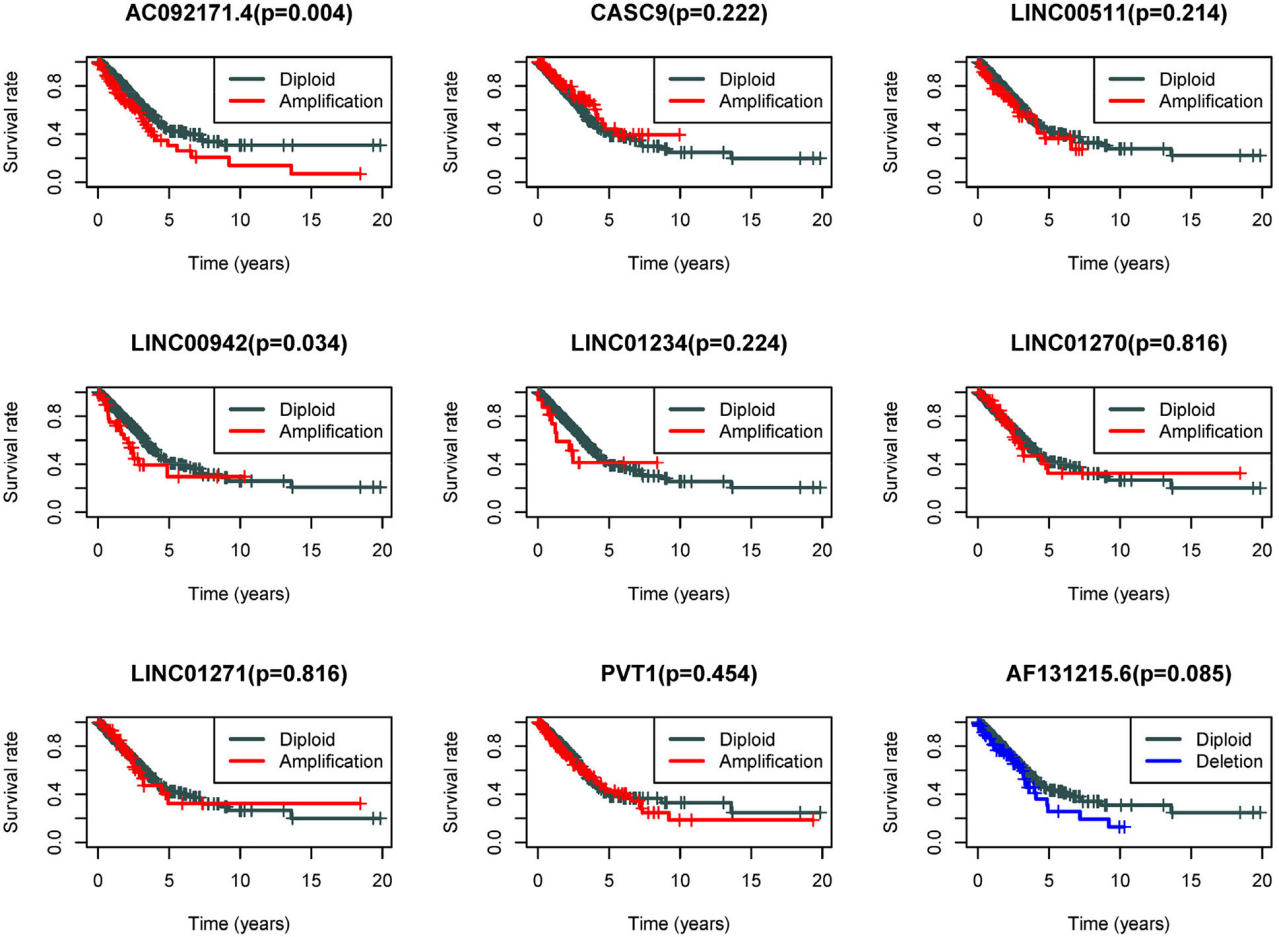
Survival analysis of the 9 lncRNAs in CNV-associated ceRNA network. Gray line represents the normal group, red line represents patients with amplification CNVs, and blue line represents patients with deletion CNVs.

Because the CNV-associated ceRNA network was built, the relationship between CNV in coding genes of lncRNA and lncRNA differential expression was simply plotted (Figure 7). The results displayed that changes in copy number of lncRNA coding genes were basically the same as the changes in expression levels. Among the nine lncRNAs in the network, there were eight high-expressed lncRNAs driven by copy number amplification and one low-expressed lncRNA driven by copy number deletion. Subsequently, survival analysis was performed using the corresponding clinical data in TCGA. The analysis results illustrated that CNVs of most lncRNAs did not directly affect the survival time of patients, whereas patients with copy number amplification in coding genes of AC092171.4 and LINC00942 had shorter overall survival time (Figure 8).

### Expression of Copy Number Variation-Driven Differentially Expressed Long Non-coding RNAs May Affect the Effect of Drug Therapy

Finally, based on previous studies, lncRNAs related to drug treatment of LUAD patients were searched in the CNV-associated ceRNA network. It was found that there were two lncRNAs (LINC00511 and LINC00942) in the CNV-associated ceRNA network that were associated with cancer response to drugs (Table 2). As revealed, LINC00511 was related to FK866 and KIN001-055, whereas LINC00942 was related to AZD8055, Bexarotene, BEZ235, GDC0941, HG-5-88-01, LFM-A13, and PD-0332991.

**Table 2:** Drugs associated with lncRNAs in the ceRNA network.

**Gene ID**	**Ensemble ID**	**Drug**	**Predictive score**	**Category**
LINC00511	ENSG00000227036	FK866	0.515	Metabolism
LINC00511	ENSG00000227036	KIN001-055	0.315	Other
LINC00942	ENSG00000249628	AZD8055	0.745	TOR signaling
LINC00942	ENSG00000249628	Bexarotene	0.285	other
LINC00942	ENSG00000249628	BEZ235	0.525	PI3K signaling
LINC00942	ENSG00000249628	GDC0941	0.375	PI3K signaling
LINC00942	ENSG00000249628	HG-5-88-01	0.355	EGFR signaling
LINC00942	ENSG00000249628	LFM-A13	0.285	Other
LINC00942	ENSG00000249628	PD-0332991	0.4	Cell cycle

## Discussion

In this study, we explored a CNV-associated ceRNA network related to LUAD by analyzing changes in CNV and expression of lncRNAs, miRNAs, and mRNAs in tumor tissue of LUAD patients. On this basis, biological functions and signaling pathways that might be affected by the CNV-associated ceRNA network were analyzed, and lncRNAs related to drug therapy were further identified. Here, differential expression of lncRNAs, miRNAs, and mRNAs between tumor tissue and normal tissue was firstly explored, and the results clarified a greater difference. Previous studies showed that RNA expression in tumor tissue is dramatically different from that in normal tissue, and such huge changes can be observed in a variety of cancers, such as liver cancer and colon cancer (Chen et al., 2016; Cui et al., 2017). Such alteration of mRNAs in cancer has been studied extensively, whereas that of miRNAs and lncRNAs remains to be explored. It is now believed that the alteration of miRNAs in tumor tissue can regulate the expression of the corresponding mRNA and then promote or inhibit the occurrence and progression of cancer in various ways (Gao et al., 2015). In addition to miRNAs, lncRNAs, a type of long RNA that do not encode proteins, are also important molecules involved in intracellular regulation, and they have been considered to be related to the regulation of gene expression since they were discovered, and now, they are believed to play a regulatory role in cells by sponging miRNAs (Paraskevopoulou and Hatzigeorgiou, 2016). At present, as the research of the lncRNA–miRNA–mRNA network (ceRNA network) goes deeper, lncRNAs are considered to have an important relationship with the occurrence and progression of tumor (Wu et al., 2018). Studies found that lncRNAs may affect the therapeutic effect of drugs through the ceRNA network. For example, lncRNA homeobox A11-Antisense RNA can promote LUAD resistance to cisplatin by affecting Stat3 through influencing miR-454-3p (Zhao et al., 2018). Therefore, exploring changes in the three types of RNA is of great value to tumor therapy.

CNV is an important type of variation in cancer, and a large number of studies demonstrate that CNV can drive the occurrence and progression of cancer. For instance, copy number amplification of MAPKAPK2 is believed to elevate the prognostic risk of lung cancer patients, whereas copy number of MAPK kinase 3 is thought to increase breast cancer risk (Kuiper et al., 2010; Liu et al., 2012; MacNeil et al., 2014). Our research found that there were quite a few CNVs in cancer tissue of patients with LUAD, and many genes with CNVs were lncRNA coding genes. An existing study believes that changes in CNV-driven lncRNA expression can affect downstream gene expression, which in turn plays a physiological regulatory role (Liu et al., 2019). Therefore, here, genes with consistent changes in CNV and expression levels were screened out and considered to be CNV-driven DElncRNAs. CNV is also one of the emerging targets in cancer detection, and it is currently the most leading-edge cancer detection technology to determine the prognostic risk of patients by detecting blood circulating cell-free DNA CNVs (Peng et al., 2019). In this study, combined with differential analysis and correlation test, a total of 67 CNV-driven DElncRNAs were selected, and they were believed to be potential biomarkers closely related to cancer occurrence.

The ceRNA regulatory network is a popular biological regulatory network. Numerous studies prove that interactions of lncRNAs, miRNAs, and mRNAs can affect the occurrence and progression of cancer. In this research, nine CNV-driven DElncRNAs were found, and a CNV-associated ceRNA regulatory network, which was composed of 9 lncRNAs [AC092171.4, AF131215.6, cancer susceptibility 9 (CASC9), PVT1, LINC00942, LINC01234, LINC00511, LINC01270, and LINC01271], seven miRNAs, and 178 effector mRNAs, was established combined with several bioinformatics databases. Most of the nine lncRNAs are proven to play a vital role in the occurrence and progression of cancer. Among them, AC092171.4 is believed to promote the progression of liver cancer by sponging miR-1271 and upregulating the expression of growth factor receptor-bound protein 2 (Sun et al., 2020). LncRNA CASC9 is a widely studied lncRNA in cancer, and it is a common oncogene that can promote the growth and proliferation of cancer cells in esophageal cancer, oral cancer, rectal cancer, breast cancer, and so on (Pan et al., 2016; Liang et al., 2018; Luo et al., 2019). This study found that changes in the expression of lncRNA CASC9 may be caused by changes in DNA copy number. Similar to CASC9, PVT1 discovered earlier can promote the occurrence and progression of cancer. Studies now believe that PVT1 can stimulate the angiogenesis of non-small cell lung cancer (NSCLC) by sponging miRNA to promote the VEGF-type transduction pathway, thereby promoting lung cancer progression, and PVT1 can also promote the growth and metastasis of NSCLC through the Wnt/β-catenin axis (Mao et al., 2019; Qi and Li, 2020). LINC00942 is rarely studied in cancer, and it is believed to promote the expression of GCLC, thereby causing poor prognosis for patients (Bajic et al., 2019; Sun et al., 2019). LINC01234 is considered to be related to the drug resistance of cancer patients, and a study displayed that LINC01234 can increase the resistance of cancer cells to chemotherapeutic drugs by regulating the LINC01234/miR-31-5p/MAGEA3 axis (Chen et al., 2020). For LINC00511, it can combine with EZH2 and LSD1 *in vivo*, downregulate the expression of LATS2 and KLF2, and promote the progression of NSCLC (Zhu F. Y. et al., 2019). LINC01271 is believed to promote the diagnostic effect of breast cancer by regulating the expression of TNS1, and it is a potential target for breast cancer treatment (Chang et al., 2020). Interestingly, most of the lncRNAs screened in this study are with copy number amplification/deletion, which causes poor prognosis for patients. Previous studies are linking CNV to the good prognosis of patients. For example, a study of colorectal cancer by Bi et al. (2019) found that the copy number amplification of SKP2 reduces the prognostic risk of colorectal cancer patients. The reason for the present study results, we reasoned that, possibly is the strict screening conditions. In addition, we also screened out seven miRNAs that play a role in the ceRNA network: miR-130b-5p, miR-30a-5p, miR-30a-3p, miR-30c-2-3p, miR-218-5p, miR-1-3p, and miR-145-3p. Most of these miRNAs are proven to be related to the occurrence and progression of various cancers. For instance, miR-130b-5p is believed to promote the proliferation and migration of gastric cancer cells (Chen et al., 2018). MiR-30a-5p is also related to the progression of various cancers (Editors, 2020; He et al., 2020). The results mentioned earlier indicate that the screened ceRNA regulatory network in this study is closely related to the occurrence and progression of cancer, and these nine key lncRNAs are related to the prognosis of lung cancer.

Based on the results mentioned earlier, enrichment analyses were conducted on downstream mRNAs, and the results revealed that these genes were mainly related to cell division and DNA repair. These two pathways are common in cancer that can promote the growth and proliferation of cancer cells (Icard et al., 2019; Zhu F. Y. et al., 2019). Besides, based on previous studies, it was found that lncRNA LINC00511 in the CNV-associated ceRNA network screened in this study was related to the treatment response of FK866 and KIN001-055, and lncRNA LINC00942 was related to the treatment response of AZD8055, Bexarotene, BEZ235, GDC0941, HG-5-88-01, LFM-A13, and PD-0332991. AZD8055, Bexarotene, BEZ235, etc., are all effective antitumor drugs, and most of these drugs are related to the mammalian target of rapamycin (mTOR) pathway and phosphatidylinositol-3-kinase (PI3K) pathway (Talpur et al., 2002; Manara et al., 2010; Willems et al., 2012). Among them, AZD8055 is an inhibitor of mTORC1 and mTORC2, and it is now believed that AZD8055 has antitumor activity in acute myeloma leukemia (Willems et al., 2012); BEZ235 is a PI3K/mTOR inhibitor that is believed to suppress the growth of tumor cells with PI3K mutations (Serra et al., 2008). The results mentioned earlier demonstrate that LINC00942 is probably related to the PI3K/mTOR pathway. The interaction between lncRNAs and drugs is an emerging research direction. With the foundation of the research discussed, cancer medication can be guided by the results of the CNV test, and the therapeutic effect on cancer of nucleic acid drugs that target lncRNAs can be enhanced based on the results of the CNV test as well (Jiang et al., 2020).

Viewed in toto, the present research was conducted on the CNV, lncRNA, miRNA, and mRNA data of TCGA-LUAD database to establish a ceRNA regulatory network related to CNV-driven DElncRNAs, and this network was also proven to be related to cell division and DNA Repair. Further analysis also disclosed that these CNV-driven DElncRNAs were related to drug efficacy. The analysis results of this study were rigorous and reliable, whereas this study is purely a bioinformatics analysis without any experimental demonstration. Thus, more biochemical and clinical experiments are needed to support the results of this study to be applied to cancer treatment.

## Data Availability Statement

The original contributions presented in the study are included in the article/Supplementary Material, further inquiries can be directed to the corresponding author/s.

## Ethics Statement

All authors consent to submit the manuscript for publication.

## Author Contributions

HH, HX, and FL contributed to the study design. EC conducted the literature search. JZ acquired the data. XL wrote the article. SX performed data analysis and drafted. HJ revised the article. QZ gave the final approval of the version to be submitted. All authors contributed to the article and approved the submitted version.

## Conflict of Interest

The authors declare that the research was conducted in the absence of any commercial or financial relationships that could be construed as a potential conflict of interest.

## Supplementary Material

Supplementary Table 1 Information on patient's clinical characteristics.

Supplementary Table 2 CNV-driven DElncRNAs screened out via correlation analysis.
